# Prospective study of the prevalence and co-morbidities of obstructive sleep apnea in active-duty army personnel in the three southernmost provinces of Thailand using questionnaire screening

**DOI:** 10.1186/s40779-018-0186-1

**Published:** 2018-11-07

**Authors:** Anisong Pilakasiri, Prasit Mahakit

**Affiliations:** Department of Otolaryngology, Phramongkutklao Hospital and College of Medicine, 315 Ratchawithi Road, Ratchathewi District, Bangkok, 10400 Thailand

**Keywords:** Active-duty army personnel, Laryngopharyngeal reflux, Prevalence, Questionnaire, Snoring, Obstructive sleep apnea

## Abstract

**Background:**

It is crucial for the army to know the prevalence of obstructive sleep apnea (OSA) syndrome in active-duty army personnel. Little information has been reported on the prevalence of OSA and clinical features in active-duty army personnel. This study was aimed to estimate the prevalence of snoring and risk of developing OSA in active-duty army personnel in Thailand and to identify the co-morbidities of OSA. In total, 1107 participants who were aged 20–60 years and were deployed to the three southernmost provinces of Thailand were enrolled. All the participants completed the Phramongkutklao (PMK) Hospital OSA Questionnaire that was modified and validated from the Berlin Questionnaire and underwent physical examination. The participants were 1107 active-duty army personnel in the three southernmost provinces of Thailand, both males and females, aged 20–60 years.

**Methods:**

The PMK OSA Questionnaire was used to assess the risk of OSA together with interviewing for snoring, fatigue, falling asleep and day-time sleepiness. Physical examination of the neck, chest and hip circumference, and height was performed. Information concerning physical training, co-morbid diseases, smoking and alcoholic consumption was collected.

**Results:**

The prevalence of snoring was 58.5, and 4.8% met the PMK OSA Questionnaire criteria, thus indicating a high risk of OSA. The information obtained indicated that laryngopharyngeal reflux (LPR), current smoking and alcoholic consumption were significantly higher in the high-risk OSA group.

**Conclusion:**

Early detection and treatment of OSA in active-duty army personnel are imperative. Physical examination and polysomnography can be used to reveal the high-risk group. High body mess index (BMI), laryngopharyngeal reflux, current smoking and alcoholic consumption are modifiable factors for OSA and are avoidable. A policy to decrease the BMI and risk of LPR, as well as to stop smoking and alcoholic consumption, should be applied.

## Background

Snoring, one of the most common sleep disorders, may affect daily activities, including personality, social and possibly marital life. For soldiers, it may affect roommates and the mission because snoring can be heard by enemies. More importantly, if snoring occurs concurrently with apnea, it may cause the reduction in the efficacy of work, increasing the risk of traffic accidents, reduce mechanical control and affect military duty. Ultimately, sleep apnea may affect heart function and sexual ability, and it may cause hypertension [1–9]. Snoring together with sleep apnea may lead to obstructive sleep apnea (OSA) syndrome. OSA may result in both short- and long-term effects. Short-term effects include morning headaches due to CO_2_ retention, tiredness, daytime sleepiness, decreased cognitive function (thinking, memory, mood or concentration) and may involve psychomotor function that may lead to accidents in traffic and working with machines [10]. Long-term effects include hypertension, cardiomegaly, especially on the right side of the heart from pulmonary hypertension, arrhythmia from the lack of oxygen, polycythemia and ischemic heart disease [11, 12].

Sharma et al. (2006) [13] studied the risks for OSA syndrome in Indian patients using the modified Berlin questionnaire. Among a group of 180 adults of middle age, 80 were at high risk whereas the other 100 were not. They concluded that conducting the modified Berlin questionnaire prior to polysomnography could identify high-risk individuals, avoiding unnecessary polysomnography. In 2006, Sharma et al. [14] showed that the prevalence rates of OSA and OSA syndrome were 13.74 and 3.57%, respectively, in semi-urban Indian citizens aged 30–60 years residing in a community. The US population was also studied by Hiestand et al. (2006) [15]. They conducted a similar study using the Berlin questionnaire in 1506 American citizens and reported that 895 (59%) had snoring problems, and 26% were at risk for OSA. In a Japanese study of 2208 males, the prevalence of severe (apnea-hypopnea index (AHI) ≥30) OSA in the studied subjects was 6.1%. Both systolic and diastolic blood pressures were significantly increased in subjects with severe OSA compared with those without OSA [16].

No concrete evidence exists on the prevalence of OSA and snoring in the Thai population. However, studies have been conducted in Thailand concerning OSA and snoring. In 2001, Anuntaseree et al. [17] performed a study on the prevalence of habitual snoring in 1142 children and its associations with tonsillar size, allergic rhinitis, obesity, parental smoking and the prevalence of OSA. They reported that the prevalence rates of habitual snoring and OSA were 8.5 and 0.69%, respectively. Additionally, the tonsillar size and allergic rhinitis were associated with snoring while only one snoring child had OSA.

The other report involving OSA and risk factors in Thailand was performed by Kongsomboon and Neruntarat [18]. They used the Berlin questionnaire in 307 subjects recruited from fourth- to sixth-year medical students at the Faculty of Medicine, Srinakarinwirot University, Thailand and showed that the prevalence of OSA in Thai medical students was 6.8%. Moreover, the subjects with BMI > 23 kg/m^2^ together with underlying diseases were at high risk. However, age, gender, academic year and academic achievement were not associated with OSA.

A study on the prevalence of OSA syndrome in the young military population in South Korea was performed by Lee et al. [19] using the modified Berlin questionnaire. They recruited 665 soldier participants (aged 20–23 years) who visited the hospital for regular checkup and found that the prevalence of snoring and a high risk of OSA syndrome were 13.5 and 8.1%, respectively. In 2013, 110 US military personnel who returned from combat within 18 months of deployment met the diagnostic criteria for OSA that was as high as 62.7%. Among these US military personnel, 70.9% returned from a combat within 12 months, and military personnel with comorbid insomnia and OSA were significantly more likely to meet the criteria for depression and PTSD [8]. In 1300 UK military personnel, the prevalence rates of snoring and OSA were 19.5 and 2.9% [6], respectively, and no study of OSA and snoring in the Thai military officer has been reported. Thus, the present study intends to investigate these aspects in the target group of the Royal Thai Army personnel.

## Methods

This is a descriptive cross-sectional study using the questionnaire created by the Health and Sleep Questionnaires of the Sleep Center, Department of Otolaryngology, Phramongkutklao Hospital, Royal Thai Army Medical Department, Thailand. This questionnaire was modified and translated from the Berlin Questionnaire and was called the PMK OSA Questionnaire (see Appendix I), which was validated for accuracy [20–26].

The Berlin Questionnaire screens for the risk of OSA. It is classified into 3 positive categories. A total score of 2 or more points from items 1–5 is reported as category 1. A total score of 2 or more points from items 6–9 is reported as category 2. A response to item 10 of ‘Yes’ or a BMI greater than 25 kg/m^2^ is reported as category 3. High risk is indicated if there are 2 or more categories where the score is positive. Low risk is indicated if there is only 1 or no category with a positive score [20–26].

The target group was the Royal Thai Army personnel working in the three southernmost provinces of Thailand. The personnel from the three task forces willing to be volunteers were arranged by age. The calculated number was 372. If up to 50% of participants could not provide appropriate answers, which equaled 186 individuals, this number was added to the sample (372 + 186); therefore, the sample size would be at least 560.

First, random sampling of active-duty Royal Thai Army personnel in the three southernmost provinces of Thailand who agreed to answer the questionnaires without any coercion was performed. Second, questionnaire explanation to personnel was performed. Next, the personnel answered the questionnaires. Thereafter, the battalion surgeon and paramedics performed the physical examination, including body weight measurement by weight scale (kg), height measurement by height scale (cm) and the circumference of the neck, chest and waist measurement (inch) by tape measure. Analyses of the data from questionnaires and physical examination reports were carried out. Volunteers were informed of the results with recommendation. Whenever the interpretation of questionnaires showed even 1 of 3 categories, it was regarded as showing risks for OSA and that personnel would be contacted for confirmation by polysomnography.

### Inclusion and exclusion criteria

Inclusion criteria were being personnel of the Royal Thai Army on active duty in the three southernmost provinces of Thailand with an age ranging from 20 to 60 years and who were willing to be enrolled. The participants also had no previous history of neck surgery, a nasal mass, chronic nasal obstruction and a deviated nasal septum.

Exclusion criteria were being previously diagnosed with upper airway obstruction, previously diagnosed with head and neck cancer, previously diagnosed with craniofacial malformation or abnormalities, congenital abnormalities, personnel reluctant to answer the questionnaires and illiterate personnel.

### Operational definition

The PMK OSA Questionnaire, which was modified from the Berlin Questionnaire, classifies an individual into 2 types, habitual snorer and high risk for OSA. The habitual snorer is an individual with a snoring frequency ≥ 4 days per week with the high risk for OSA displaying either laryngopharyngeal reflux or allergic rhinitis or both. The common symptoms of laryngeal reflux are hoarseness, throat pain, sensation of a lump in the throat, cough, repetitive throat clearing, excessive phlegm, and difficulty in swallowing, heartburn and voice fatigue [27]; the common symptoms of allergic rhinitis are recurrent episodes of sneezing, pruritus, rhinorrhea, nasal congestion and lacrimation occurring after exposure to the offending allergen [28].

In Thailand, BMI is classified as follows: < 18.5 = underweight, 18.5–22.9 = normal, 23.0–24.9 = risk of becoming overweight, 25.0–29.9 = obesity type 1, > 30 = obesity type 2. This study defines the term “overweight” for a subject with BMI > 23. This includes the risk of becoming overweight and both obesity type 1 obesity type 2 [29].

### Data collection

The data were collected from October 2013 to September 2014 in Narathiwat province, and the collected data were analyzed at the Department of Otolaryngology, Phramongkutklao Hospital, Royal Thai Army Medical Department, Thailand. This study protocol was reviewed and approved by the Institutional Review Board of the Royal Thai Army, Medical Department (R129q/57). Informed consent for participation and publication was obtained and signed from all participants according to the Declaration of Helsinki.

### Statistical analysis

The information from the questionnaires and physical examinations were analyzed using a statistical program. Descriptive statistics such as number, percentage, mean, standard deviation, minimum and maximum were reported. Categorical data were analyzed using chi-squared test, while continuous data were analyzed by independent *t*-test. Logistic regression analysis was used to identify variables independently correlated with OSA syndrome. The results were presented as the means ± SD or as adjusted odds ratios and corresponding 95% confidence intervals. Statistical significance was inferred at *P* <  0.05.

## Results

The data obtained showed the mean body weight increased from 64.6 kg to 65.6 kg during a one-year period. The means of the height and body mass index were 169.6 cm and 22.7 kg/m^2^, respectively, while the circumference values of the neck, chest and waist were 15.3, 42.5 and 31.9 in., respectively.

More details of the data are displayed in Table 1. The 1107 subjects included 1036 males and 71 females with a mean age of 26.9 ± 8.0 years. These Royal Thai Army personnel included three different groups: 596 (53.8%) privates, 408 (36.8%) non-commissioned officers and 25 (2.3%) commissioned officers (Table 1).

**Table 1:** Prevalence of obstructive sleep apnea analyzed with various risk factors

Item	*n*	%	OSA (*n*(%))	*P* value	Snoring (*n*(%))	*P* value
Gender				0.382^a^		0.453
Male	1036	93.6	48 (4.7)		497 (48.9)	
Female	71	6.4	5 (7.0)		31 (44.3)	
Age (year)				0.009^a^		0.001
< 30	812	74.1	34 (4.2)		366 (45.7)	
30–39	202	18.4	9 (4.5)		110 (55.6)	
40–49	45	4.1	4 (9.5)		28 (70.0)	
> 50	37	3.4	6 (16.7)		23 (63.9)	
BMI(kg/m^2^)				0.05		< 0.001
< 25	865	79.5	34 (3.96)		376 (44.34)	
25–30	187	17.2	14 (7.53)		119 (65.03)	
30	36	3.3	5 (13.89)		27 (75.00)	
Rank				0.058^a^		0.074
Private	596	53.8	24 (4)		280 (47.7)	
NCOs	408	36.8	19 (4.7)		204 (50.5)	
COs	25	2.3	3 (12.0)		17 (68.0)	
Hypertension				0.008^a^		0.002
Negative	1056	98.2	44 (4.2)		496 (47.7)	
Positive	19	1.8	4 (21.1)		16 (84.2)	
Diabetes				1.000^a^		0.500†
Negative	1074	99.8	48 (4.5)		512 (48.4)	
Positive	2	0.2	–		–	
Dyslipidemia				< 0.001^a^		< 0.001
Negative	1042	96.9	41 (3.9)		486 (47.4)	
Positive	33	3.1	8 (24.2)		26 (78.8)	
Allergic Rhinitis				0.224		0.226
Negative	950	88.4	39 (4.1)		447 (47.9)	
Positive	125	11.6	8 (6.5)		66 (53.7)	
Laryngopharyngeal reflux				< 0.001		< 0.001
Negative	729	71.1	22 (3.0)		301 (41.9)	
Positive	296	28.9	29 (9.8)		197 (67.5)	
Smoking				0.579		0.001
Never	427	40.3	22 (5.2)		176 (41.7)	
Quit	125	11.8	8 (6.5)		61 (50.8)	
Still smoking	508	47.9	22 (4.3)		272 (54.1)	
Piece/day^c^	11.5 ± 7.5	10 (1–78)	13.8 ± 7.8	0.140^b^	11.5 ± 6.7	0.545‡
Alcoholic drinking				0.007		< 0.001
Never	393	45.4	10 (2.5)		144 (37)	
Quit	164	18.9	14 (8.6)		89 (55.6)	
Still drinking	309	35.7	15 (4.9)		177 (57.7)	
Glasses/day^c^	5.3 ± 5.2	3 (1–30)	8.3 ± 7.8	0.046^b^	5.3 ± 4.9	0.213‡

*NCOs*: Non-commissioned officers; *COs*: Commissioned officers; *OSA*: Obstructive sleep apnea

Chi-Squared test

^a^Fisher’s exact test

^b^Mann-Whitney U test

^c^Presented as means ± SD and medians (Min-Max)

It was found that the prevalence rates of snoring and OSA obtained by the PMK OSA questionnaire were 58.5 and 4.8%, respectively.

From univariate analysis (Table 2), the risk factors for OSA were increasing age, BMI, hypertension, diabetes mellitus, dyslipidemia, laryngopharyngeal reflux (LPR), alcoholic consumption and alcoholic drinking (8.3 ± 7.8 glasses/day), while the risk factors for snoring were increasing age, hypertension, dyslipidemia, LPR, smoking, alcoholic consumption and alcoholic drinking (5.3 ± 4.9 glasses/day).

**Table 2:** Multiple logistic regression

Item	*n*	%	OSA (*n*(%))	Crude *OR*	95%CI	*P* value	Adjusted *OR*	95% CI	*P* value
Gender									
Male	1036	93.6	48 (4.7)	1			1		
Female	71	6.4	5 (7.0)	1.55	0.6–4.02	0.369	1.90	0.38–9.38	0.433
Age (year)									
< 30	812	74.1	34 (4.2)	1			1		
30–39	202	18.4	9 (4.5)	1.08	0.51–2.28	0.847	0.66	0.16–2.73	0.571
40–49	45	4.1	4 (9.5)	2.41	0.81–7.13	0.113	0.99	0.12–7.97	0.994
> 50	37	3.4	6 (16.7)	4.57	1.78–11.72	0.002	6.44	0.96–43.12	0.055
Rank									
Private	596	53.8	24 (4)	1			1		
NCOs	408	36.8	19 (4.7)	1.17	0.63–2.16	0.627	0.74	0.22–2.45	0.655
COs	25	2.3	3 (12.0)	3.24	0.91–11.59	0.070	2.66	0.32–21.93	0.111
BMI (kg/m^2^)									
< 25	865	79.5	34 (3.96)	1			1		
25–30	187	17.2	14 (7.53)	1.98	1.04–3.76	0.038	1.25	0.47–3.33	0.655
> 30	36	3.3	5 (13.89)	3.91	1.43–10.69	0.008	3.17	0.77–13.11	0.11
Hypertension									
Negative	1056	98.2	44 (4.2)	1			1		
Positive	19	1.8	4 (21.1)	6.12	1.95–19.19	0.002	0.66	0.07–6.15	0.712
Dyslipidemia									
Negative	1042	96.9	41 (3.9)	1			1		
Positive	33	3.1	8 (24.2)	7.79	3.31–18.32	< 0.001	4.06	0.80–20.53	0.090
Allergic rhinitis									
Negative	950	88.4	39 (4.1)	1			1		
Positive	125	11.6	8 (6.5)	1.62	0.74–3.55	0.229	1.19	0.41–3.42	0.749
Laryngopharyngeal reflux									
Negative	729	71.1	22 (3.0)	1			1		
Positive	296	28.9	29 (9.8)	3.5	1.98–6.2	< 0.001	4.27	1.99–9.17	< 0.001
Smoking									
Never	427	40.3	22 (5.2)	1			1		
Quit	125	11.8	8 (6.5)	1.28	0.55–2.95	0.566	0.80	0.24–2.64	0.712
Still smoking	508	47.9	22 (4.3)	0.83	0.45–1.52	0.550	0.36	0.13–0.98	0.046
Alcoholic drinking									
Never	393	45.4	10 (2.5)	1			1		
Quit	164	18.9	14 (8.6)	3.62	1.57–8.34	0.002	6.17	1.99–19.14	0.002
Still drinking	309	35.7	15 (4.9)	1.95	0.87–4.41	0.107	3.58	1.14–11.21	0.029

*NCOs:* Non-commissioned officers; *COs*: Commissioned officers; *OSA*: Obstructive sleep apnea

From multivariate analysis (Table 2), LPR, smoking and a history of previous alcoholic consumption and current alcoholic consumption were strong and significant risk factors for OSA. However, old age (> 50 years), BMI and rank showed no statistical significance with OSA.

## Discussion

This study was the first in adults with a large sample size and the first to investigate the Royal Thai Army personnel on active duty in the three southernmost provinces of Thailand.

The prevalence of OSA in our study was 4.8% while that in young male Korean soldiers was 8.1% [19]. The cause may be that the prevalence of overweight in Korean individuals is higher than that in Thai individuals based on the WHO global database on BMI. For the male population in the Republic of Korea (ROK), the percentage of overweight was 32.4–36.6%, while that in Thailand was 20.9–22.6%. Among the young male Korean soldiers, only privates were recruited; however, in our study, we recruited commissioned officers (COs), non-commissioned officers (NCOs) and privates of both male and female genders aged 20–60 years to participate in the study. Additionally, among the US and UK male population, the overweight percentages were 61.6–72.1% and 66.3–67%, respectively. These findings might explain why our prevalence was less than that in other studies.

In a Japanese study, 2208 males were enrolled, similar to our situation where males predominated. In that study, the prevalence rates of mild-to-moderate (5 ≤ AHI < 30) and severe (AHI ≥ 30) OSA were 7.1 and 6.1%, respectively [16]. Although the number of recruited males outnumbered that of females, similar to our study, the Japanese prevalence rate was higher. This was probably due to their recruitment of 18- to 69-year-old males with a mean age of 44.4 ± 0.2 years, which was higher than that in our study.

Regarding the 1506 American citizen study [15], 26% were at risk for OSA, but our study had a lower number of subjects at risk for OSA because of the differences in the mean age and percentage of females in each study. In 2013, 110 US military personnel who returned from a combat within 18 months of deployment were recruited in the study and showed that 62.7% of them met the diagnostic criteria for OSA. Additionally, 97.3% of these US military personnel were men with a mean age of 33.6 ± 8.0 years and a mean BMI of 30.0 ± 4.3 kg/m^2^. Moreover, 70.9% of them returned from a combat within 12 months, and military personnel with comorbid insomnia and OSA were significantly more likely to meet the criteria for depression and PTSD [20]. Although 93.6% of the participants were male in our study, they had a lower mean age (26.9 ± 8 years) and lower mean BMI (22.7 ± 3.2 kg/m^2^) and were still on active duty.

A study on the prevalence of snoring and sleep-disordered breathing among military personnel in the UK was conducted in 1300 personnel [6]. They also recruited subjects of different ranks and genders, and the percentage of females was low (11.3%), similar to that in our study (6.4%). The prevalence rates of snoring and OSA were 19.5 and 2.9%, respectively, while those in our study were 58.5 and 4.8%, respectively. Our study with a higher percentage of snoring and OSA might have resulted from them being on active duty, whereas the studied military personnel in the UK were at regular military bases located in England. Active duty may cause stress and tiredness, leading to snoring and OSA.

In one study on the prevalence of OSA in Thai medical students [18] using the Berlin questionnaire, 6.8% of subjects indicated a high risk for OSA, higher than that reported in our study (4.8%). The cause might be that the mean age of our study was 26.9 ± 8.0 years while that in Thai medical students was 21.9 ± 1.0 years. Additionally, the percentage of females in our study was only 6.4% while that of Thai medical students was as high as 61.8%. The difference in age and the gender ratio might cause different results.

The risk factors for OSA from our study were LPR, smoking and a history of previous alcoholic consumption and current alcoholic consumption. In terms of the association between LPR and OSA, in our study, the prevalence of LPR was 28.9%, and it was a strong risk factor for OSA, corresponding to the study of Kongsomboon and Neruntarat (2006) [18] who stated that co-morbidities, especially peptic ulcer and GERD, were risk factors for OSA.

Increasing age showed a trend associated with OSA, but there was no significance. This likely occurred because our population was not at normal distribution and most of the participants were young males.

Smoking was a risk factor for OSA, showing a *p*-value of 0.046, which corresponded to that in the study in 2016 by Appleton in 1011 Australian adults aged ≥18 years. Among this population, 49.8% were male and the prevalence of OSA diagnosed by an overnight sleep study was 8.3%. The diagnosed and possible undiagnosed OSA and simple snoring were independently associated with male gender, obesity, and current smoking. The odds ratio of current smoker and diagnosed OSA and possible undiagnosed OSA were 3.2 and 3.2, respectively [30].

Alcohol consumption was associated with a higher risk of sleep apnea, corresponding to the results of a meta-analysis published in 2018 [31] and those of our study. Regarding multivariate analysis, the adjusted odds ratio of subjects who quit alcoholic drinking (5.84 and *P* = 0.002) was higher than that of subjects who were still drinking (3.13 and *P* = 0.044). The cause is likely because the subjects who quit alcoholic drinking might have had some medical conditions that motivated them to quit drinking and these medical conditions could be risks for OSA. Further identification of these medical conditions is needed.

From our study, we found other factors that might cause the deviation—for example, when the participants slept alone, they could not tell whether they snored. Additionally, participants might not understand the questionnaire or answer the questionnaire truthfully. Sometimes, they could have co-morbidities of which they were unaware. In our study, we collected the data from both male and female active-duty army personnel aged 20–60 years, but the number of males was predominant; therefore, it could not represent the population because of differences in terms of age, gender and occupation. However, we can apply these questionnaires as a screening tool for the risk of the OSA. However, polysomnography could be used as a gold-standard diagnosis.

In addition to further study to obtain more accurate data, it is suggested that all 53 positive tests should be compared with 53 negative tests and that all participants should be subjected to polysomnography, which is the gold standard.

## Conclusion

The prevalence rates of snoring and a high risk of OSA in active duty army personnel were 58.5 and 4.8%, respectively. Although these values were lower than those of previous studies, snoring and OSA among military personnel is an agenda that needs to be administered seriously. Each individual at risk needs to be referred to a specialist for further investigation and treatment urgently. BMI, LPR, smoking and alcoholic consumption were modifiable risk factors for OSA. Weight control and tailored exercise for each subject should reduce and prevent LPR and OSA, which have common risk factors and may result in more efficient work performance. The policy to stop smoking and alcohol intake is of potential therapeutic and preventive value in this condition.

## Abbreviations

BMI: Body mass index
COs: Commissioned officers
LPR: Laryngopharyngeal reflux
NCOs: Non-commissioned officers
OSA: Obstructive sleep apnea
PMK: Phramongkutklao
PTSD: Posttraumatic stress disorder
ROK: Republic of Korea
